# Prevalence and Characteristics of Fibromyalgia in Patients with Foot and Ankle Pain: The Experience of an Academic Podiatry Clinic

**DOI:** 10.3390/medicina59010058

**Published:** 2022-12-27

**Authors:** Jacopo Ciaffi, Lorenzo Brognara, Giacomo Gangemi, Elena Vanni, Elisa Assirelli, Simona Neri, Giulia Casadei, Antonio Mazzotti, Alberto Di Martino, Cesare Faldini, Stefano Zaffagnini, Francesco Ursini

**Affiliations:** 1 Medicine & Rheumatology Unit, IRCCS Istituto Ortopedico Rizzoli (IOR), 40136 Bologna, Italy; elena.vanni97@gmail.com (E.V.); elisa.assirelli@ior.it (E.A.); simona.neri@ior.it (S.N.); francesco.ursini2@unibo.it (F.U.); 2 Podiatry Teaching Clinic of the University of Bologna, IRCCS Istituto Ortopedico Rizzoli (IOR), 40136 Bologna, Italy; lorenzo.brognara2@unibo.it (L.B.); giacomo.gangemi@studio.unibo.it (G.G.); 3 Department of Biomedical and Neuromotor Sciences (DIBINEM), University of Bologna, 40125 Bologna, Italy; antonio.mazzotti@ior.it (A.M.); albertocorrado.dimartino@ior.it (A.D.M.); cesare.faldini@ior.it (C.F.); stefano.zaffagnini@ior.it (S.Z.); 4 Medical Clinic of Doctor Accorsi, 40123 Bologna, Italy; giuly.casadei@gmail.com; 5 1st Orthopaedic and Traumatologic Clinic, IRCCS Istituto Ortopedico Rizzoli (IOR), 40136 Bologna, Italy; 6 2nd Orthopaedic and Traumatologic Clinic, IRCCS Istituto Ortopedico Rizzoli (IOR), 40136 Bologna, Italy

**Keywords:** fibromyalgia, prevalence, epidemiology, podiatry, foot, ankle

## Abstract

*Background and Objectives:* Research about the prevalence of fibromyalgia in podiatric patients is limited, with data suggesting potentially higher estimates and greater foot impairment in patients with fibromyalgia compared to healthy individuals. The aim of our study is to assess the prevalence of fibromyalgia in the podiatric healthcare setting and to research the characteristics of fibromyalgia patients with foot or ankle disorders. *Materials and Methods*: Consecutive patients visiting the academic podiatry clinic at the University of Bologna IRCCS Rizzoli Orthopaedic Institute between 11 January and 31 March 2021 were enrolled. *Results*: Of the 151 patients included, 21 met the fibromyalgia survey diagnostic criteria, accounting for a prevalence of 13.9% (95% CI 8.8–20.5). As part of the podiatric assessment, the Foot Function Index (FFI) was used to calculate the impact of foot and ankle problems. Moreover, patients with fibromyalgia were asked to complete the fibromyalgia impact questionnaire (FIQ). Fibromyalgia patients had significantly worse total FFI scores (63.4 ± 23.0% vs. 53.2 ± 20.3%, *p* = 0.038) and there was a significant linear correlation between the FFI and the FIQ (r = 0.72, *p* < 0.001). *Conclusions*: The prevalence of fibromyalgia in the academic podiatry clinic being 13.9% confirms that, in the healthcare setting, the disease can be more frequent than in the general population. Furthermore, our findings suggest a strong correlation between foot impairment and the impact of fibromyalgia.

## 1. Introduction

Fibromyalgia (FM) is a common rheumatic disorder mainly characterized by chronic widespread pain, sleep disturbances, and fatigue [1]. The pathogenesis of FM remains largely unknown [2] but patients complain about a wide range of symptoms, including gastrointestinal disorders, stiffness, cognitive impairment, headache, psychological suffering, autonomic disturbances, gait instability, and hypersensitivity to external stimuli, suggesting that peripheral and central mechanisms might be involved. Moreover, emerging evidence shows that FM patients can have impaired stability, balance deficiencies, and difficulties in maintaining an upright posture, which is possibly associated with sensory deficits and disorders of the vestibular system.

The prevalence of FM is estimated to be between 2% and 4% worldwide, with a strong female predominance of 3:1 [3] and higher proportions in risk groups, such as patients affected by obesity [4] or autoimmune rheumatic diseases [5]; however, figures may diverge significantly according to the applied diagnostic criteria [6]. In Italy, different prevalence data have been reported, ranging from 3.6% to 6.6% [7,8]. In the healthcare setting, FM was found to be more frequent, showing a prevalence of 13.4% in tertiary care university hospital pain clinics [9], 15% in patients admitted in internal medicine wards [10], and as high as 33% in hospital-visiting individuals [11].

To our knowledge, the only article assessing the prevalence of FM in the podiatric population was published by Harvey in 1993 [12]. In this study, Harvey reported an overall figure of 2.3%, with estimates of 20% in patients with plantar heel or arch pain. Therefore, he concluded that higher percentages of FM could be found in podiatric patients and that particular attention should be paid to patients with pain in the plantar area; however, no other characteristics of this group were highlighted. Few other studies focused on finding foot-related features that may be unique to FM patients. A case–control study by Padín-Galea et al. [13] showed that the frequency of stiffness and mobility impairments or the presence of foot lesions, such as hyperkeratosis, were comparable in FM patients and healthy subjects, though FM patients more often complained of foot or ankle pain of significantly higher intensity and consequently needed to use more pain killers. Similarly, Palomo-López et al. assessed foot health status in women who suffered from FM using the Foot Health Status Questionnaire (FHSQ) and demonstrated that FM patients had worse foot health-related quality of life compared to healthy women [14].

Taken together, these data suggest that FM patients might experience foot and ankle problems more often than healthy individuals, but also that the prevalence of FM in foot care clinics might be higher than in the general population. However, the prevalence of FM in podiatric patients has not been re-evaluated in recent years. The aim of our study is to investigate the epidemiological aspects of FM in individuals visiting the podiatry clinic of our center and to analyze their clinical characteristics.

## 2. Materials and Methods

### 2.1. Study Design and Population

We conducted a cross-sectional, observational study of consecutive patients attending the academic podiatry clinic at the University of Bologna IRCCS Rizzoli Orthopaedic Institute, Bologna, Italy (hereinafter the “Institution”). Patients were included in the study if they were 18 years of age or older, visited the clinic between 11 January and 31 March 2021, and had acute or chronic foot pain. Exclusion criteria were foot or ankle surgery within the last year, conditions causing major gait disorders such as amputation, neuromuscular diseases or hip dysplasia, recent injury to ligaments of the foot or ankle, and neurocognitive impairment. Patients visiting the podiatry clinic repeatedly during the study period were considered only at the time of the first visit. Demographic characteristics and data about comorbidities were recorded. Patients were then examined in two subgroups based on the presence of a diagnosis of FM according to the validated Italian version of the FM survey diagnostic criteria (FSDC) [15,16,17] and compared in terms of demographic characteristics, comorbidities, the usage of foot orthoses, the presence of chronic foot pain, the need for paracetamol or non-steroidal anti-inflammatory drugs (NSAIDs), the chief complaint, the main involved area, and the impact of foot and ankle pathology.

### 2.2. Podiatric Assessment

All patients underwent a comprehensive assessment by two podiatrists with more than 10 years of experience (L.B. and G.C.). For the purpose of the study, we collected data about the chief complaint leading the patient to seek attention from the podiatrist along with information regarding the affected foot or ankle and the current use of orthoses. Information regarding the presence of chronic foot pain (lasting more than 3 months) and the need to regularly use (at least twice a week in the last 3 months) paracetamol or NSAIDs were systematically recorded. As part of the podiatric evaluation, the Italian version of the Foot Function Index (FFI) [18] was given to all patients to measure the impact of foot and ankle impairment. The FFI is a patient-reported outcome originally developed for patients with rheumatoid arthritis, but its reliability and validity have been established in several populations [19,20]. Since the index has demonstrated high versatility in several fields [21], it has been widely used in foot and ankle research, including rheumatological observational studies [22] and studies of orthotics or other medical interventions [23]. The revised version [18] is composed of 17 questions divided into three categories: pain (5 questions), disability (9 questions), activity limitation (3 questions). Each question is scored from 0 to 10. Scores are added and then divided by the maximum total possible. Unanswered questions are excluded from the computation. Decimal points are eliminated by multiplying the score by 100. A total score and scores for each subscale can be obtained. The psychometric properties of the Italian version of the FFI are acceptable [24]. Reliability is satisfactory for the pain and disability subscales. Validity has shown strong correlation between FFI subscales and the items of the SF-36 and pain VAS, which measure similar constructs [24]. The Italian version of the FFI is also able to detect change over time, which should be >5.4 for the pain subscale and >9.3 for the disability subscale to be considered significant [24].

### 2.3. Fibromyalgia Survey

To define the presence of FM, the validated Italian version of the FM survey diagnostic criteria (FSDC) was applied [15,16,17]. This questionnaire has been used in epidemiological studies, demonstrating reliability and convergent and discriminant validity [25]. The FSDC is a self-administered tool with 3 sections. In the first part, patients are asked to use a 0–3 Likert scale to rate the intensity of fatigue, cognitive problems, and unrefreshing sleep during the past week, accounting for a maximum score of 9. The next section includes 3 yes or no questions investigating whether participants experienced abdominal pain or cramps, depression, and headache over the past 6 months, providing a maximum of 3 points. A symptom severity score (SS) ranging from 0 to 12 is obtained by adding the scores of sections 1 and 2. In the last part of the questionnaire, patients identify which sites were perceived as painful or tender during the last week out of 19 possible body areas (left and right shoulder girdle, left and right upper arm, left and right lower arm, left and right hip, left and right upper leg, left and right lower leg, left and right jaw, chest, abdomen, upper back, lower back, and neck), resulting in a 0–19 widespread pain index (WPI). The sum of the WPI and SS gives a fibromyalgianess scale (FS) ranging from 0 to 31, with a score ≥ 13 used as the cut-off point to distinguish FM cases [16].

Patients meeting the FSDC were asked to complete the Italian version of the FM impact questionnaire (FIQ) [26]. The FIQ is one of the most used tools in evaluating FM patients and has shown good test–retest reliability and construct validity [26]. It is composed of 10 items assessing physical function, workplace absenteeism, occupational impairment, pain, fatigue, morning tiredness, stiffness, anxiety, and depression. The overall score ranges from 0 to 100, with higher values indicating greater impact from the disease. Regarding the psychometric properties of the Italian version of the FIQ, test–retest reliability is between 0.74 and 0.95 for physical functioning, total score, and other components. Overall internal consistency is 0.90. FIQ items are significantly correlated with the HAQ and SF-36, indicating good construct validity [26,27]. Although it has not been assessed in Italian, other language versions of the questionnaire have been shown to be responsive to perceived clinical improvement [28,29].

### 2.4. Statistical Analysis

Based on data available from previous studies regarding FM epidemiology in the healthcare setting, we hypothesized the prevalence of FM in podiatry clinics to be about 10%. Accordingly, we calculated a minimum sample size of 139 patients to estimate such a proportion with a 5% margin of error and 95% confidence interval. Data are expressed as a mean (standard deviation) or a number (percentage), as appropriate. An independent sample Student’s t-test was used to compare differences in normally distributed continuous variables between patients with FM and controls. Fisher’s exact test was used to compare categorical variables. The Clopper–Pearson “exact” [30] method was used to calculate a 95% confidence interval (95% CI) of FM prevalence based on the beta distribution. Pearson’s correlation coefficient was used to assess the association between the FIQ and FFI scores in the FM group. A *p*-value of <0.05 was considered statistically significant. All analyses were performed using Statistical Package for Social Sciences (SPSS) software version 26.0 (IBM).

### 2.5. Ethical Considerations

This study was conducted in compliance with the Declaration of Helsinki and its latest amendments [31]. All patients were asked to sign a written informed consent form before participation. The study was approved by the local ethics committee (Comitato Etico Area Vasta Emilia Centrale, Bologna, Italy—approval number: 659/2021).

## 3. Results

### 3.1. Characteristics of the Included Population and the Prevalence of Fibromyalgia

During the study period, 151 adult patients were evaluated in the academic podiatry clinic of the Institution. Mean age was 66.3 (16.5) years, and 101 individuals (66.9%) were women. Average BMI was 25.8 (4.8) kg/m². A total of 11 patients (7.3%) used custom-made foot orthoses, 76 subjects (50.3%) suffered from chronic pain (lasting over 3 months), and 40 (26.5%) reported the need for paracetamol or NSAIDs to relieve their foot symptoms. The most common chief complaint that led to consulting a podiatrist was the presence of a callus (53.6%), followed by an ingrown toenail (28.5%). The toes (30.5%), the toenails (27.2%), and the forefoot (25.8%) were the main areas involved. Diabetes (19.2%) and osteoporosis (14.6%) were the most represented comorbidities. Assessing the impact of foot disorders in the included patients, mean total FFI was 54.6 (20.9), while the subscale scores for pain, disability, and activity limitation were 60.3 (21.8), 59.1 (26.3), and 31.3 (22.4), respectively. General characteristics of the included population are shown in Table 1.

Twenty-one individuals had an FSDC score of ≥13, accounting for an overall FM prevalence of 13.9% (95% CI 8.8–20.5). In the FM group, 16 patients were women and 5 were men, resulting in an FM prevalence of 15.8% (95% CI 9.3–24.4) in women and 10% (95% CI 3.3–21.8) in men. The characteristics of FM patients are reported in Table 2. The mean FIQ score in FM patients was 52.2 (22.6). The average scores for each of the 10 FIQ items are shown in Table 3. Analyzing the association between FIQ and FFI scores, a significant linear correlation emerged between the FIQ and the total FFI (r = 0.72, *p* < 0.001), between the FIQ and FFI pain subscale (r = 0.68, *p* = 0.001), between the FIQ and FFI disability subscale (r = 0.72, *p* < 0.001), and between the FIQ and FFI activity limitation score (r = 0.46, *p* < 0.036). Furthermore, when analyzing FM prevalence according to the duration of foot pain, prevalence was 26.3% (95% CI 16.9–37.7) in patients with foot pain lasting >3 months and 1.3% (95% CI 0–7.2) in patients with symptoms lasting <3 months.

### 3.2. Differences between Patients with or without Fibromyalgia

No significant difference was observed in demographic characteristics, chief complaint, the involved foot or ankle area, or the presence of comorbidities regardless of whether patients met the FSDC or not. The proportion of individuals with foot pain lasting more than 3 months was higher in FM than in non-FM (95.2% vs. 43.1%, *p* < 0.001), and the use of paracetamol and NSAIDs was more frequent in FM patients (52.4% vs. 22.3%, *p* = 0.007). Evaluating the different impact of foot problems in the two groups, FM patients had significantly worse total FFI scores (63.4 ± 23.0% vs. 53.2 ± 20.3%, *p* = 0.038). Similarly, the subscale scores for pain (70.3 ± 18.9% vs. 58.7 ± 21.9%, *p* = 0.024) and activity limitation (56.3 ± 23.0% vs. 27.3 ± 19.6%, *p* < 0.001) were also significantly higher in individuals with FM. Differences between patients with or without FM are reported in Table 2.

## 4. Discussion

Although FM and foot problems such as corns, calluses, ingrown toenails, athletes foot, bunions, veruccas, and diabetic foot are common conditions in the general population [1,32,33,34,35,36], there is only one article, published in 1993, reporting data about the prevalence of FM in podiatric patients [12]. Intrigued by the ability to provide updated prevalence estimates of FM in the foot care setting, we conducted a study of 151 patients in an academic podiatry clinic. We found an overall prevalence of 13.9% and higher numbers in women than in men, which is in line with studies describing FM as a predominantly female disease. Our results also confirm that, in the healthcare setting, FM can be substantially more common than in the general population [9,10,11,37], particularly in patients with chronic complaints; this is suggested by our finding that a higher prevalence of FM can be observed in individuals with foot pain lasting for longer periods of time.

To our knowledge, this research may be considered as the first study applying the FFI and the FIQ to FM patients recruited from a podiatry clinic. Using these tools, we evaluated the impact of foot pathology on function, and we found that FM patients had a significantly higher FFI than non-FM individuals. We observed a strong linear correlation between each FFI domain and overall FIQ score, suggesting a bidirectional relationship between the impact of FM on patients and their foot problems. These results are consistent with the findings of Palomo-López et al. that demonstrated that FM patients had worse quality of life related to foot problems compared to healthy controls [14]. It is conceivable that, also in FM patients visiting the academic podiatry clinic, foot and ankle pain is a significant limitation for the activities of daily living, thus leading to physical function deterioration due to reduced mobility and the perception of worsened quality of life.

In a case–control study, Padín-Galea et al. [13] showed that FM patients more often complained of foot or ankle problems and needed more pain killers than healthy subjects. Consistently, patients with FM visiting our clinic had long-lasting foot pain more frequently than non-FM individuals, resulting in a significantly greater use of analgesics. Notwithstanding the use of paracetamol or NSAIDs, FFI pain scores were still higher in FM patients, suggesting that foot problems do not respond adequately to this therapeutic approach, at least in patients with FM.

Given the high prevalence and the substantial economic burden of FM [38], these findings could help to establish new tools for early detection and more tailored treatment of foot and ankle disorders in FM patients, with a significant impact on public health in general. For FM patients, it is important to correctly identify foot and ankle problems in order to prompt an effective treatment strategy. As demonstrated in our study, foot and ankle pain is strongly correlated with the severity of FM and can be a major source of activity limitation. Foot and ankle disorders may also impair the possibility of engaging in physical activity, which is recommended in FM to reduce pain and improve quality of life [39,40]. However, in FM, psychological factors also play a major role and are deeply interconnected with physical determinants [41]. Evidence suggests that addressing factors such as catastrophizing psychological distress and physical fatigue is crucial to reducing the severity of FM, while resilience allows patients to maintain function despite ongoing stress, similar to what happens in other rheumatic diseases [41,42,43,44].

In order to identify a possible screening tool for early detection of FM patients in the podiatric population, we evaluated several studies assessing different walking parameters, in which the existence of gait alterations in FM patients was suggested [45,46,47]. According to the literature, individuals affected by FM have a higher double support phase and stance phase but walk with lower velocity and a shorter cadence, stride length, swing phase, and single support phase compared to healthy controls [45,48]. Therefore, gait analysis can be used to identify FM patient subgroups [46], though there is no consensus about the need to screen for the presence of FM during daily podiatry practice.

Our findings support the idea that an FM screening tool in the podiatric setting may be useful to identify whether foot complications could be related to FM and consequently prompt a multidisciplinary approach when needed. Furthermore, our study suggests that foot pain and reduced quality of life experienced by the patient may be largely related to FM rather than caused by the foot or ankle problem itself.

Despite providing estimates about the prevalence of FM in the podiatry setting, our study has some limitations to acknowledge. First, the design is cross-sectional. Although it is conceivable that treatment efficacy might be different in FM patients, we could not evaluate the outcomes of podiatry interventions. The strong correlation between the FIQ and FFI suggests that the effectiveness of treatments might potentially be impaired in individuals with poorly controlled FM symptoms, and also suggests that improvements in FM symptoms might lead to fewer foot and ankle problems. Prospective research with a longitudinal design is warranted to clarify the role of podiatric treatment in FM patients. Secondly, the limited number of FM cases precludes the ability to perform adequately powered sub-analyses on patients with distinct characteristics and makes it challenging to determine whether other factors contribute to the severity of foot and ankle symptoms experienced by the FM group. Therefore, studies with a larger population would be needed to further explore the topic and possibly confirm our results. We also acknowledge that individuals visiting a podiatry clinic do not necessarily represent the general population of individuals with foot and ankle problems. This may have resulted in selection bias in the study sample. However, in order to minimize this risk, unselected patients were enrolled to avoid overly restrictive exclusion criteria.

## 5. Conclusions

For the first time in three decades, we explored the epidemiology of FM in the podiatric setting, where we found a high prevalence of the disease. Using the FFI and the FIQ, we showed a strong correlation between the impact of FM and foot problems, and we also demonstrated that foot complaints are more severe in FM patients that in non-FM individuals. Prospective research with a longitudinal design is warranted to clarify the role of podiatric care in order to ameliorate FM symptoms and functionality in these patients.

## Figures and Tables

**Table 1:** General characteristics of the patients included in the study.

Characteristics	Patients (*n* = 151)
Women, *n* (%)	101 (66.9)
Age, mean (SD)	66.3 (16.5)
BMI, mean (SD)	25.8 (4.8)
Current smoking, *n* (%)	14 (9.3)
Previous (>1 year) foot or ankle surgery, *n* (%)	15 (9.9)
Use of foot orthoses, *n* (%)	11 (7.3)
Foot pain lasting > 3 months, *n* (%)	76 (50.3)
Use of paracetamol or NSAIDs, *n* (%)	40 (26.5)
**FSDC ≥ 13, *n* (%)**	21 (13.9)
**Chief complaint**	
Callus, *n* (%)	81 (53.6)
Ingrown nail, *n* (%)	43 (28.5)
Other, *n* (%)	27 (17.9)
**Main involved area**	
Toes, *n* (%)	46 (30.5)
Nails, *n* (%)	41 (27.2)
Forefoot, *n* (%)	39 (25.8)
Other, *n* (%)	25 (16.5)
**Comorbidities**	
Diabetes, *n* (%)	29 (19.2)
Hypertension, *n* (%)	13 (8.6)
Peripheral neuropathy, *n* (%)	8 (5.3)
History of stroke, *n* (%)	2 (1.3)
History of cancer, *n* (%)	8 (5.3)
Chronic kidney disease, *n* (%)	2 (1.3)
Osteoporosis, *n* (%)	22 (14.6)
Rheumatoid arthritis, *n* (%)	9 (6)
**FFI**	
FFI total score, mean (SD)	54.6 (20.9)
FFI pain score, mean (SD)	60.3 (21.8)
FFI disability score, mean (SD)	59.1 (26.3)
FFI activity limitation score, mean (SD)	31.3 (22.4)

BMI: body mass index; FFI: foot function index; FSDC: fibromyalgia survey diagnostic criteria; SD: standard deviation.

**Table 2:** Differences between patients with or without FM.

	Patients with FM (*n* = 21)	Patients without FM (*n* = 130)	*p*-Value
Women, *n* (%)	16 (76.2)	85 (65.4)	0.455
Age, years, mean (SD)	68.8 (11.8)	65.8 (17.1)	0.445
BMI, kg/m^2^, mean (SD)	25.8 (5.8)	25.8 (4.6)	0.882
Current smoking, *n* (%)	0	14 (10.8)	0.220
Previous (>1 year) foot or ankle surgery, *n* (%)	4 (19)	11 (8.5)	0.134
Use of foot orthoses, *n* (%)	3 (14.3)	8 (6.2)	0.183
Foot pain lasting > 3 months, *n* (%)	20 (95.2)	56 (43.1)	<0.001
Use of paracetamol or NSAIDs, *n* (%)	11 (52.4)	29 (22.3)	0.007
**Chief complaint**			
Callus, *n* (%)	14 (66.7)	67 (51.5)	0.242
Ingrown nail, *n* (%)	3 (14.3)	40 (30.8)	0.191
Other, *n* (%)	4 (19.0)	23 (17.7)	1
**Involved area**			
Toes, *n* (%)	6 (28.6)	40 (30.8)	1
Nails, *n* (%)	3 (14.3)	38 (29.2)	0.192
Forefoot, *n* (%)	8 (38.1)	31 (23.8)	0.184
Other, *n* (%)	4 (19.0)	21 (16.2)	0.754
**Comorbidities**			
Diabetes, *n* (%)	4 (19)	25 (19.2)	1
Hypertension, *n* (%)	1 (4.8)	12 (9.2)	0.695
Peripheral neuropathy, *n* (%)	1 (4.8)	7 (5.4)	1
History of stroke, *n* (%)	1 (4.8)	1 (0.8)	0.260
History of cancer, *n* (%)	3 (14.3)	5 (3.8)	0.082
Chronic kidney disease, *n* (%)	0	2 (1.5)	1
Neurodegenerative disorders, *n* (%)	0	1 (0.8)	1
Osteoporosis, *n* (%)	5 (23.8)	17 (13.1)	0.195
Rheumatoid arthritis, *n* (%)	1 (4.8)	8 (6.2)	1
**FFI**			
FFI total score, mean (SD)	63.4 (23.0)	53.2 (20.3)	0.038
FFI pain score, mean (SD)	70.3 (18.9)	58.7 (21.9)	0.024
FFI disability score, mean (SD)	61.9 (29.1)	58.7 (26.0)	0.613
FFI activity limitation score, mean (SD)	56.3 (23.0)	27.3 (19.6)	<0.001
**FIQ score, mean** (**SD**)	52.2 (22.6)		

BMI: body mass index; FFI: foot function index; FIQ: fibromyalgia impact questionnaire; FM: fibromyalgia; SD: standard deviation.

**Table 3:** Items of FIQ score.

FIQ Item	Mean (SD)
Physical functioning	6.5 (2.8)
No days felt good	7.8 (2.2)
Workdays missed	6.5 (3.0)
Ability to do job	5.2 (2.8)
Pain	4.8 (2.8)
Fatigue	4.6 (3.0)
Tiredness	4.3 (3.1)
Stiffness	4.8 (3.2)
Anxiety	3.7 (2.8)
Depression	4.1 (3.0)
**Total FIQ**	52.2 (22.6)

FIQ: fibromyalgia impact questionnaire.

## Data Availability

All relevant data are reported within the manuscript.
